# Strain-dependent structure and Raman behaviours in the heavy-ion irradiated manganite at extreme low dose

**DOI:** 10.1038/s41598-019-55638-1

**Published:** 2019-12-16

**Authors:** Nam Nhat Hoang, Duc Huyen Yen Pham, The Nghia Nguyen

**Affiliations:** 1 Laboratory of Low Dimensional Materials and Applications, Faculty of Engineering Physics and Nanotechnology, VNU-University of Engineering and Technology, 144 Xuan Thuy, Cau Giay, Ha Noi 10000 Viet Nam; 2 Department of Physics, Chungbuk National University, Cheongju, 28644 South Korea; 3 Department of Nuclear Physics, Faculty of Physics, VNU-University of Science, 334 Nguyen Trai, Thanh Xuan, Ha Noi 10000 Viet Nam

**Keywords:** Nanoscale materials, Structural materials, Techniques and instrumentation, Theory and computation

## Abstract

The microstrains in heavy-ion irradiated manganite LaMnO_3_ can be managed in linear response of irradiation dose, and the corresponding internal pressure up to 8 GPa can be induced by varying doses. The response of structure under stress is studied by means of Density Functional Theory and Lattice Dynamic Calculation. All obtained Raman scattering lines are discussed in details to shed light onto structural changes during ion implantation. There appears new resonance peak at around 550 cm^−1^, which splits from broad features in the spectra, and attributes to the anti-symmetric vibrations of O_6_ cages. The blue shift of this peak scales to ~2.4 cm^−1^ per 1 GPa of stress. Another strong feature showing considerable blue shift is seen in the vicinity of 640 cm^−1^ and corresponds to one of rhombohedral distortion related soft modes. A weak mode, not frequently reported, is seen at around 420 cm^−1^ and corresponds to translation-like motions of fixed O_6_ cages.

## Introduction

The LaMnO_3_ (LMO) has attracted the attention of scientists for both fundamental and application researches for decades due to a number of important effects that coexist within a single structure frame. Among the most interesting ones are colossal magnetoresistance^1^, magnetic and orbital orderings^2,3^, insulator-metal transition and ferromagnetism^4,5^. Many of these are believed to have their origins in the double exchange interaction between Mn^3+^−O−Mn^4+^ pairs, which is accompanied by Jahn-Teller distortion of coordination octahedra of Mn ions^6^. Although the nominal oxidation state of Mn in LMO is 3+ (electronic configuration ${t}_{2g}^{3}{e}_{g}^{1}$) there always exists a portion of Mn in 4+ state (${t}_{2g}^{3}{e}_{g}^{0}$) due to non-stoichiometric oxygen content often presented, so the ferromagnetic double exchange interaction is a typical phenomenon in this compound. It is important to note here that the strength of Jahn-Teller gap (≈2 eV) falls within a range of energy covered by Raman resonances, therefore the Raman spectroscopy appears essential for the study of effects arising from magnetic orderings in LMO. Indeed, the early studies^2,7^ have proposed that the orbital orderings may be observed in Raman at a few hundreds cm^−1^. The first classification of Raman scattering bands for LMO in orthorhombic space group *Pnma* (no. 62) was given quite early^8^, and later the same group of authors also reported the classification for the higher symmetry *R*$\bar{3}$*c* (no. 167)^9^. Basically, LMO exhibits two different structures depending on sintering temperature: the low temperature orthorhombic *Pnma* and the high temperature rhombohedral *R*$\bar{3}$*c*. Both are derived from parent cubic lattice *Pm*$\bar{3}$*m* (no. 221). The Raman spectra of these two structures are very similar and possess broad features near 300, 500 and 600 cm^−1^, but the assignments of modes in the two cases are different. By measuring the Raman scatterings in different orientations and varying temperature (in some cases from below Curie temperature *T*_*C*_ to above *Néel* temperature *T*_*N*_) the origins of each scattering bands were identified^8–11^. Some high frequency bands (above 1000 cm^−1^) were assigned to orbitons^12,13^ (that is, the excitations of orbital wave according to Franck-Condon mechanism^14^) and were claimed as the first experimental observation of orbitons. But later on they were shown to be the false assignments, as the features are originated in multi-phonons^15,16^. The importance of Raman spectroscopy in the study of LMO arises from its high sensitivity to local disorders caused by Jahn-Teller distortions of MnO_6_ octahedra. It is known that under high pressure the O_6_ cages can rotate in precession motions against each other, promoting the changes in Mn-O bond lengths and angles that can be probed by Raman. Indeed, there are a number of LMO studies involving Raman as an analytical tool^17–20^, especially where doping concentrations are low^10,21^. Since the internal pressure can be induced by lattice strains, it is reasonable to question whether or not the strains can be engineered (at ambient pressure) and observed simultaneously for LMO. In this paper we show that the microstrains can be smoothly varied by heavy-ions bombardments, so that the internal pressure is linearly induced, and observed by both X-Ray and Raman measurements. To rule out the impacts associated with changing oxygen stoichiometry we are working only in a region of concentrations that are much lower than the usual oxygen non-stoichiometric content. Our LMO samples are irradiated at extremely low doses by LaO^−^ high energy ion beam, resulting in deeply pierced materials of certain density of doped sites. The focus is paid on how the stress created by heavy-ion insertion influences the structure and Raman behaviours of the compounds.

## Results and Discussion

### Structure under stress

**Figure 1:**
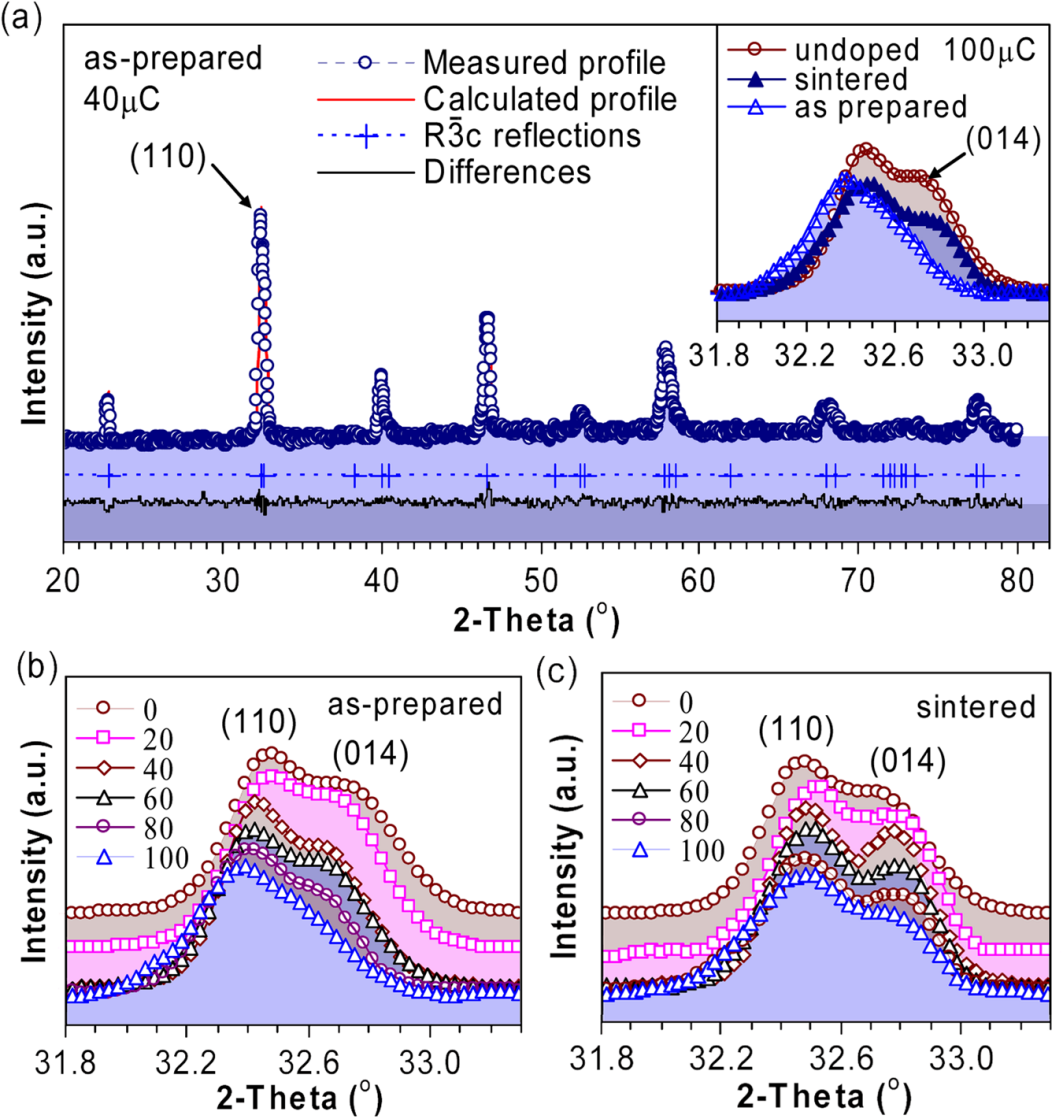
X-ray diffractograms of irradiated samples. (**a**) The measured and calculated profiles for as-prepared 40 μC irradiated sample; the inset compares the profiles of the strongest reflection before and after sintering for 100 μC sample. The profiles of the strongest reflection are shown for (**b**) as-prepared and (**c**) sintered samples.

**Figure 2:**
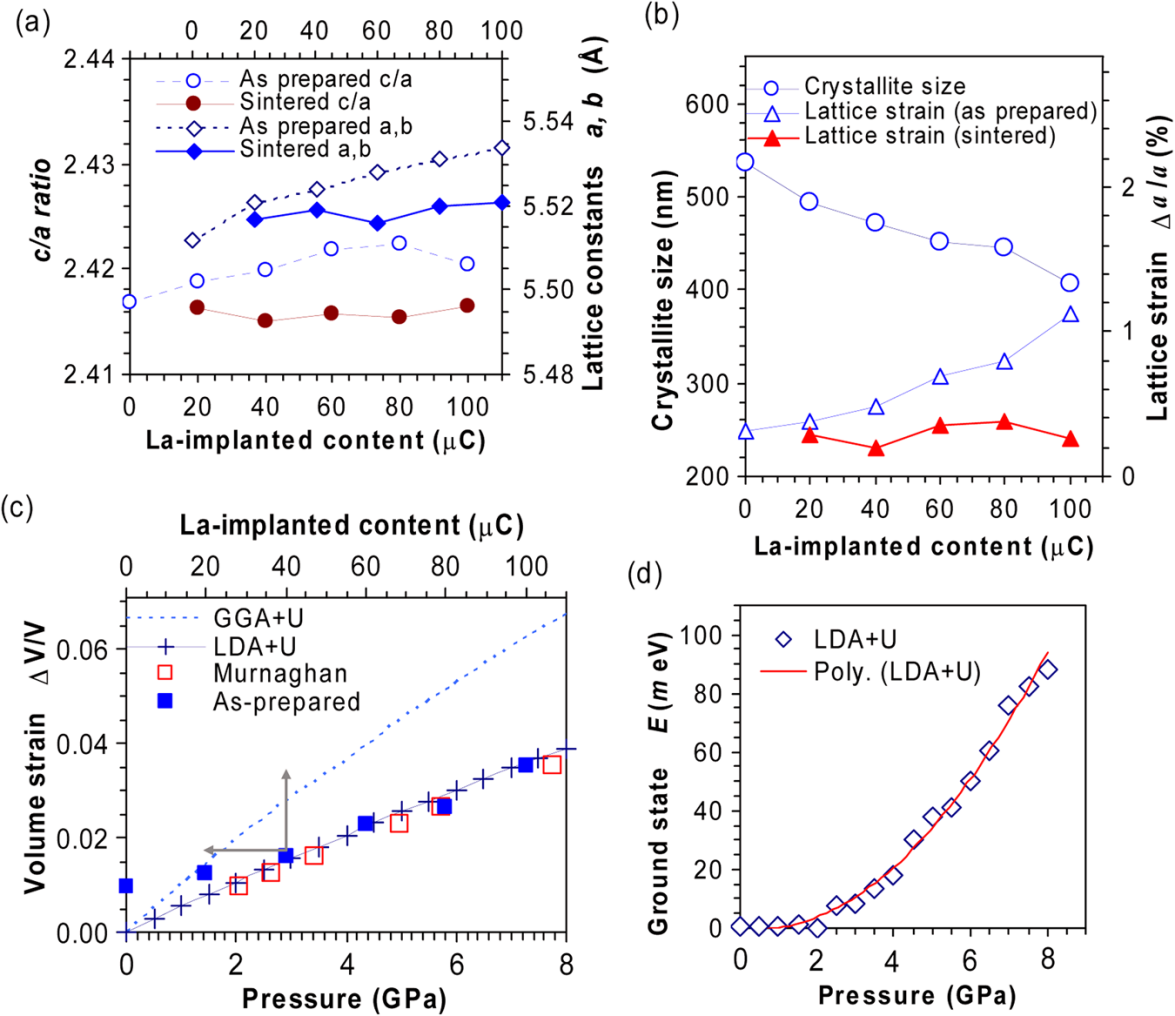
Lattice constants, crystallite sizes and strains of irradiated samples. (**a**) The lattice constants for as-prepared and sintered samples; (**b**) The crystallite sizes and lattice strains; (**c**) LDA+U calculated external pressure and Murnaghan’s empirical pressure ($\Delta V/V=1-{e}^{-P/c}$)^26^; (**d**) The increase of LDA+U ground state energy upon pressure (polynomial fitting is given).

Because LaO^−^ doses are low (<0.05% of La-content in bulk samples), the associated oxygen non-stoichiometry should be minimal. So, we assume that any non-stoichiometric content should be located at the interstitial sites and contributed to the strains. The Rietveld refinements were carried out for all samples in *R*$\bar{3}$*c* space group with optimization for the following variables: lattice parameters (*a, c*), site occupation factors (La, Mn, O), thermal motions, *x* coordinates of oxygen atoms. Corrections for backgrounds, preferred orientations, zero points were applied. For the determination of crystallite sizes and lattice strains the modified Thompson-Cox-Hastings profiles^22^ were used. The obtained results are summarized in Table 1. As the typical diffractogram, the one obtained for the as-prepared 40 μC irradiated sample is shown in Fig. 1(a), together with the inset which enlarges the strongest reflection before and after sintering for another sample (100 μC). It is obvious that there is a shoulder split from the strongest peak at around 32.78°, which is present in all as-prepared samples (Fig. 1(b)) and grows stronger in the sintered ones (Fig. 1(c)). This peak is a typical *R*$\bar{3}$*c* scattering line (014) and is neither present in the higher *Pm*$\bar{3}$*m* nor in the lower *Pnma* symmetry, whereas the strongest peak (around 32.45°), which is indexed as (110) in *R*$\bar{3}$*c*, is also seen as (011) in *Pm*$\bar{3}$*m* or as (200) in *Pnma*^23^. For the as-prepared samples, Fig. 1(b) depicts two main effects as irradiation increases: the weakening of *R*$\bar{3}$*c*-related (014) shoulder reflections and the clear shift to lower angles of (110) and (014) peaks. While the first argues for shifts of symmetry towards lower *Pnma* at higher irradiation doses, the second implies increases of lattice constants as dose increases. Table 1 shows that, there is a systematic prolongation of all axes, but this seems to be an isotropic effect with no preferred orientation as the *c/a* ratios do not change convincingly from a value of the un-doped sample. The average increase of unit cell volumes is about 1% (the largest increase is 1.35% for 100 μC sample). On the other hand, Fig. 1(c) does not show the similar changes for sintered samples, indeed the lattice parameters of all sintered samples are very close to a value of the un-doped one. A clear relaxation of lattice after sintering can also be seen in the inset of Fig. 1(a). To clarify this relaxation, Fig. 2(a) compares the lattice parameters of the as-prepared and the sintered samples. It is evident that after increases during irradiation the lattices of sintered samples compressed and relaxed to the smaller lattices, close to that of the un-doped structure. Let us consider the changes of symmetry by converting the *R*$\bar{3}$*c* cells into the pseudo-*Pnma* by using a transformation matrix [(1, 2/3, −1/3);(0, 4/3, −2/3);(0, 1/3, 1/3)]. Doing so, we obtain the *Pnma* axes of appropriate lengths, but they lose orthogonality: γ ≈ 90° (*α* = *β* = 90°). As seen in Table 1, the deviations of γ from 90° are equal for the un-doped and sintered samples (this means they remain *R*$\bar{3}$*c*) but visibly show a decrease for the as-prepared samples, which implies shifts of symmetry towards the lower *Pnma*.

**Table 1:** The results of Rietveld refinements for irradiated LMO samples (*R*$\bar{3}$*c*, no. 167, *α* = *β* = *90°*, γ = 120°).

Sample	LaO^–^ (μC)	*a* (Å)	*c* (Å)	*c/a*	|γ−90°|/90° (%)	*x*(O)	Mn-O_6_ (Å)	Mn-O_6_-Mn (°)	*R*_P_ (%)
As prepared	0	5.512 (5)	13.322 (4)	2.417	0.80	0.4521	1.958	164.5	6.3
	20	5.521 (4)	13.354 (3)	2.419	0.76	0.4497	1.967	163.4	5.9
	40	5.524 (1)	13.367 (5)	2.420	0.72	0.4452	1.969	162.3	5.6
	60	5.528 (4)	13.388 (4)	2.422	0.68	0.4436	1.972	161.8	5.8
	80	5.531 (1)	13.398 (3)	2.422	0.67	0.4422	1.974	161.3	6.0
	100	5.534 (1)	13.395 (5)	2.420	0.71	0.4400	1.977	160.6	5.3
Sintered	20	5.517 (2)	13.331 (1)	2.416	0.82	0.4431	1.969	161.6	5.3
	40	5.519 (5)	13.329 (8)	2.415	0.86	0.4440	1.967	161.9	6.5
	60	5.516 (7)	13.325 (4)	2.416	0.83	0.4452	1.965	162.2	6.1
	80	5.520 (7)	13.333 (8)	2.415	0.83	0.4416	1.969	161.2	6.6
	100	5.521 (5)	13.341 (8)	2.416	0.82	0.4420	1.968	161.3	5.2

The standard deviations are given in the parenthesis, the figure-of-merit *R*_*P*_ is for the profiles. The occupation factors of La were all refined to 1.00, whereas that of *Mn* to 0.99 and O to 0.98. The concentrations are given in the total charge of irradiated ions (μC). All six Mn-O_6_ bond lengths are equal.

The increase of unit cell volumes after irradiation is not usual but may be understood as a consequence of loading (or unloading) internal (or external) pressure. The supposed lattice strains can be directly obtained from relative increases of lattice constants *ε*_*a*_ = Δ*a/a*, *ε*_c_ = Δ*c/c*. For the as-prepared samples the average *ε*_*a*_ and *ε*_*c*_ are 0.24 and 0.37%, and for the sintered ones they reduce to 0.12 and 0.07% correspondingly. This decrease agrees well with observed relaxations after heat treatment. It is evident that, *ε*_*a*_ and *ε*_*c*_ themselves are not adequate in describing the total internal stress, as the individual lattices, including the un-doped one, are bearing their own non-uniform microstrains which attribute to the broadening Δ*θ* of diffraction lines. Putting aside the irrelevant (instrumental and crystallite size broadening), in quasi-cubic lattices the broadening caused by lattice strains is linear function of tan*θ*_*hkl*_, *i.e*. Δ(2*θ*_*hkl*_) = *ε*_*θ*_ tan*θ*_*hkl*_^24^ (assuming the Gaussian peak shape). For the analysis of profile by Rietveld technique, this type of broadening is included in the modified Thompson-Cox-Hastings (TCH) profile^22^. Reasonably, for the samples prepared under the same conditions the average broadening *ε*_*θ*_ should be close to each other. Indeed, we obtained 0.40% for the as-prepared, and 0.31% for the sintered and un-doped samples. The similar trend and close values of microstrains are often seen in the heavy-ion irradiated samples, *e.g*. in the Ag^9+^ irradiated SrTiO_3_^25^. Thus, the total lattice strain should be given as *ε* = *ε*_*a*_ + *ε*_*θ*_ . The obtained results for *ε* are shown in Fig. 2(b). It appears that the bombardment of LaO^−^ ions reduces both crystallite sizes and symmetry while simultaneously increases microstrains and unit cell volumes. Since the microstrains increase with increases of unit cell volume, it is naturally to assume that the negative pressure is induced by microstrains. This pressure may be derived from the Murnaghan’s equation^4,26^, or by modelling the structures under stress using the Density Functional Theory (DFT). Figure 2(c) compares *ε*, translated into the equivalent volume expansions ΔV/V, with calculated values obtained from DFT. In general, LaMnO_3_ is a challenging case for DFT modelling^27^ where Local Density Approximation (LDA) is known to provide good estimates of band-gap and structure, but more elaborated functionals (GGA) are also used^28,29^. To reproduce the band gaps^30^ and ground state correctly the on-site Coulomb repulsion (U parameter) and antiferromagnetic spin configuration need to be included. For our cases, the best agreement is achieved with LDA functional for U = 6.5 eV (2.8% error in band-gap, 0.9% in cell volume), whereas the deviations are large with GGA (>200% in band gap, 15% in cell volume). It is interesting to estimate how much external pressure is needed to force the same volume change as what is induced by increase of internal stress. Of course, the volume compression is not necessarily the same as the volume expansion, but we assume them to be equivalent. As seen, the dependence of ΔV/V on irradiation dose corresponds well to that of the simulated ΔV/V on external pressure. The agreement between the pressures derived from Murnaghan’s equation and LDA is also good. Particularly, 8.0 GPa increase of pressure stimulates a unit cell compression by 3.6% (while raises the ground state energy by ~ 100 *m*eV, Fig. 2(d), the relative increase is about 12.4 *m*eV/GPa), so it follows that 7.8 GPa is needed to remove the microstrains related remaining pressure (~2.0 GPa, Fig. 2(c)) and compress the cell of 100 μC irradiated sample into an equal cell of the un-doped one. These values are found also in good agreement with the previous data reported for LMO under isotropic pressure^4^. Thus, the physics here is simple: the increase of dose leads to the increase of microstrains, which in turn increases the internal stress and the expansion of volume is a consequence.

**Figure 3:**
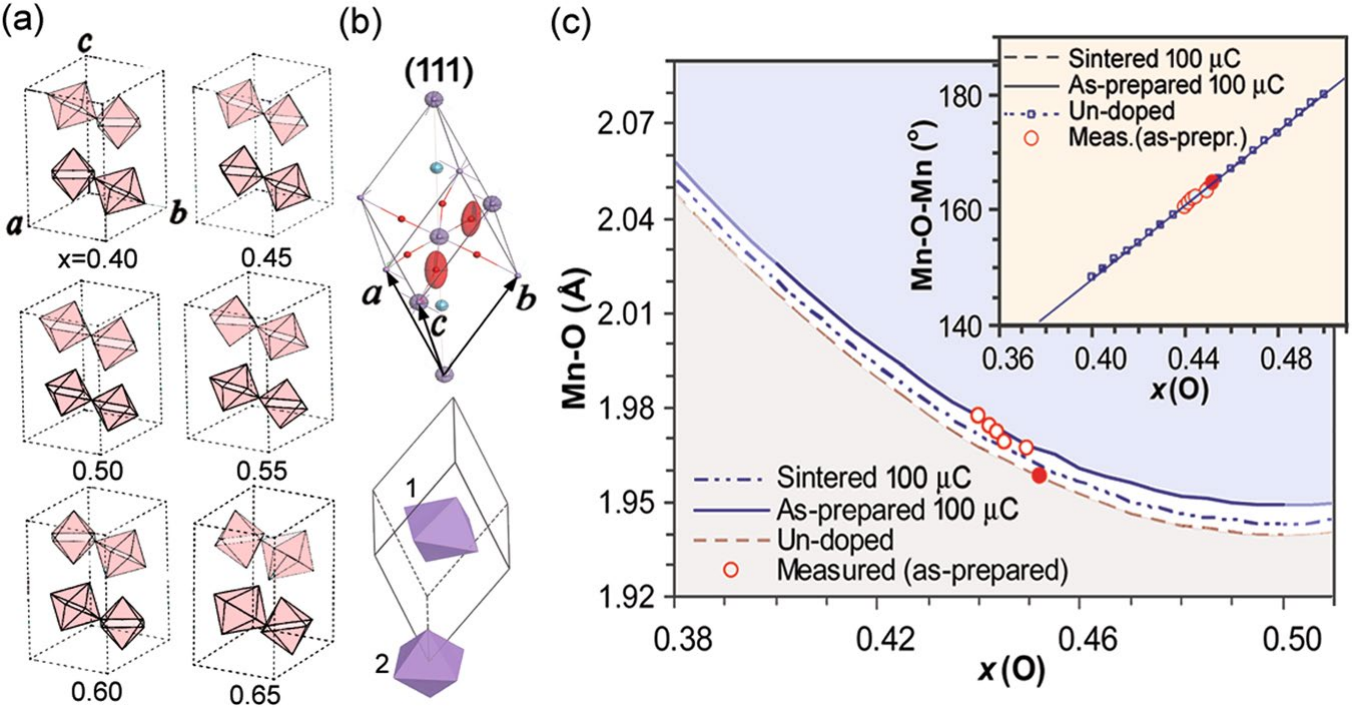
Illustration of rhombohedral distortion. (**a**) LMO in hexagonal representation: rotations of MnO_6_ octahedra as *x-*coordinates of oxygen atoms change; (**b**) LMO in a rhombohedral primitive cell: preferred movements by thermal ellipsoids, and stacking of tilted MnO_6_ octahedra; (**c**) Theoretical variations of Mn-O distance and Mn-O-Mn angle (the inset) according to *x-*coordinates of oxygen for the sintered (100 μC), as-prepared (100 μC) and un-doped cell. The measured data are also given for the as-prepared samples (red circles, the filled ones denote the un-doped case).

Now to assist the analysis of Raman spectra, we discuss the effect of stress on positions of oxygen atoms. In *R*$\bar{3}$*c* the oxygen atoms occupy the Wyckoff’s sites *18e* (*x, 0, 1/4*)^23^ with *x* ≈ 0.43. When *x* = 0.50 the structure reduces to cubic, and for *x* = 0.40 the O_6_ cages are strongly tilted, but still remain symmetric with 6 equal Mn−O bonds. The only possible crystallographic invariant deformation is the precession rotation of O_6_ cages (Fig. 3(a)). The reduced rhombohedral cells are shown in Fig. 3(b), where preferred thermal motions of oxygen atoms around the rhombohedral axis [101]_*r*_ are revealed by the shapes of thermal ellipsoids. As seen, two symmetry related O_6_ cages 1 and 2 possess different precessions and their rotation axes are parallel only in the cubic structure. The corresponding changes of Mn−O distances and Mn-O-Mn angles are shown in Fig. 3(c), together with the theoretical values deduced directly from *x*. While the Mn-O-Mn angle (related to rotation of O_6_ cages) is practically linear with *x* and is independent of cell choices, the Mn-O distance (related to breathing of O_6_ cages) shifts to higher values for the sintered and as-prepared samples. The linear change of Mn-O-Mn angle upon rhombohedral distortion (inset, Fig. 3(c)) and the corresponding linearity of the soft modes’ shifts upon pressure (Table 2) can be regarded as the good evidences for the equivalence of the actions of positive and negative pressure in low dose region.

**Table 2:** Peak positions (cm^−1^) for the un-doped *R*$\bar{3}$*c* LMO, the front sides and back sides of irradiated as-prepared samples and the sintered samples.

Assigned	As-prepared (Front sides)					As-prepared (Back sides)				
	20	40	60	80	100	20	40	60	80	100
O(*A*_*1g*_)			257	256	260					
O_6_(*E*_*g*_)	326	327	325	325	325	326	325	322	320	328
O_6_(*E*_*g*_)	417	421	420	418	419					
O(*E*_*g*_)	486	486	484	488	489	486	488	486	488	489
O(*E*_*g*_)	557	556	558	559	563	551	552	554	556	553
O(*E*_*g*_)	640	639	641	643	644	641	636	632	639	635
O(*E*_*g*_)		667					664	666	665	667
**Un-doped**	**Sintered samples (Front sides)**					**Results of other works on** ***R***$\bar{3}$***c*** **LMO**				
	**20**	**40**	**60**	**80**	**100**	ref. ^9^	^10^	^46^	^45^	^18^
						236	230	210	217	205
326	327	327	328	328	325	329	315	313	323	320
	414	411	417	414	407		435	412	427	425
486	488	487	490	490	487		495	492	497	486
544	550	541	547	557	539	520	505			
638	644	634	632	630	642	640	620	605	618	610
665		662	665	663						658

The doping contents are given in μC.

### Raman spectra of structures under stress

**Figure 4:**
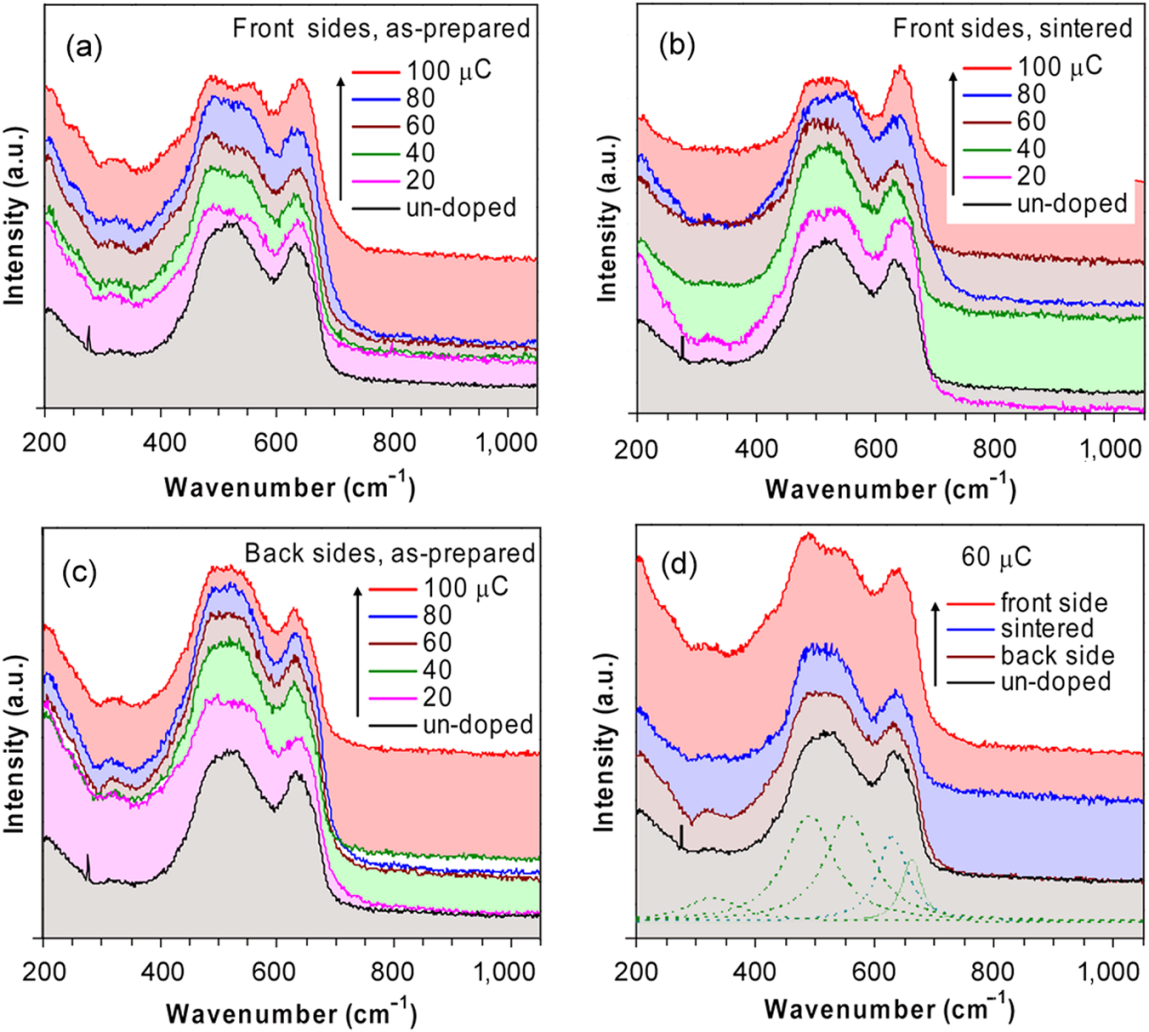
Raman spectra of polycrystalline LMO in *R*$\bar{3}$*c* recorded at room temperature. (**a**) The front sides of as-prepared samples; (**b**) The front sides of sintered samples; (**c**) The back sides of as-prepared samples; (**d**) Comparison of spectra recorded for one sample (60 μC).

The obtained Raman scattering spectra are shown in Fig. 4 for the un-doped sample, the front sides (which face against the ion beam) of the as-prepared samples (Fig. 4(a)), the back sides (which attach to the substrate) of the same samples (Fig. 4(b)), and the sintered samples (front sides, Fig. 4(c)). The comparison of spectra obtained for 60 μC irradiated sample is featured in Fig. 4(d). At the first sight, it appears that the scattering patterns of the fresh as-prepared samples are different for the front sides and the back sides. Also, the patterns from the back sides are similar to that of the un-doped sample. The similarity also appears between the un-doped and the sintered samples (Fig. 4(b)). The process of strains and relaxation is illustrated in Fig. 4(d) where one may see a split of a new peak at 558 cm^−1^ (front side) from a broadened peak at 450–600 cm^−1^ (back side). This split disappears for a sintered sample. The similarity between the un-doped and sintered samples again confirms on the absence of annealing effect caused by laser beam during Raman experiment. The obtained peaks positions are listed in Table 2. Two scattering bands at 544–563 and 638–644 cm^−1^ show the largest blue shifts which are almost linear upon doses. For the un-doped sample, the peaks are identified at 326, 486, 544 and 638 cm^−1^. The same peak structure is seen for the back sides of as-prepared samples, with some variation in frequencies: 320–328, 486–489, 551–556, and 632–641 cm^−1^. The main deviations of the front sides from the others include (*i*) clear splits of new peaks at 544–563 cm^−1^ and (*ii*) disappearances of shoulders at around 665 cm^−1^. Two bands with minor intensities are seen at 256–260 and 417–421 cm^−1^. The later is also repeated in the spectra of sintered samples (407–417 cm^−1^). Unlike in *Pnma* the Raman in *R*$\bar{3}$*c* exhibit only a few active modes due to constrains in symmetry. According to group theory^31^, the following active modes are available for each Wyckoff’s sites:

La (6a): M = Silent(*A*_*2g*_) + IR(*A*_*2u*_ + *E*_*u*_) + Raman(*E*_*g*_)

Mn (6b): M = (Hyper-Raman)*A*_*1u*_ + IR(*A*_*2u*_ + 2*E*_*u*_), (no Raman active mode)

O (18e): M = (Hyper-Raman)*A*_*1u*_ + Silent(2*A*_*2g*_) + IR(2*A*_*2u* _+ 3*E*_*u*_) + Raman(*A*_*1g*_ + 3*E*_*g*_)

**Figure 5:**
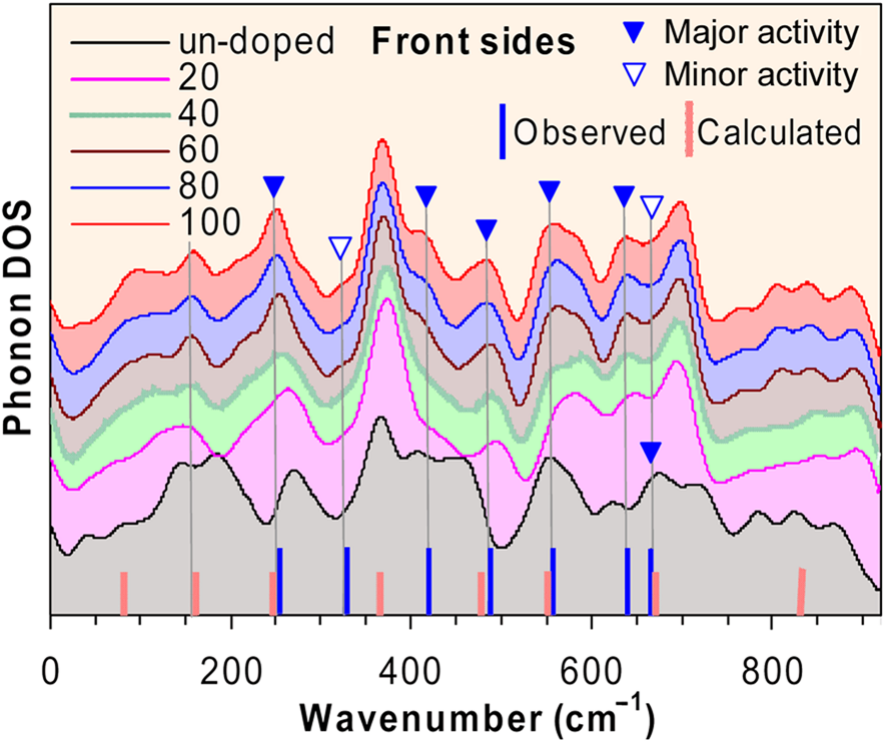
Calculated phonon density of states (PDOS) for implanted samples. Filled (blank) triangles denote major (minor) activities. Observed and calculated Raman activities in *R*$\bar{3}$*c* are also shown.

Therefore, a final mechanical representation of Raman contains only 1*A*_*1g*_ and 4*E*_*g*_ modes. The previous lattice dynamic calculation^9^ revealed the *A*_*1g*_ at 249 cm^−1^ (assigned to O_6_ rotation around hexagonal [001]_*h*_ axis), one oxygen-related *E*_*g*_ modes at 468 (bending vibration) and another at 646 cm^−1^ (out-of-phase stretching vibration). The assignment of *A*_*1g*_ mode to O_6_ rotation is consistent with our analysis of precession rotations of O_6_ cages (Fig. 3), so it seems to be unambiguous that 256–260 cm^−1^ peaks observed in 80 and 100 μC as-prepared samples attribute to *A*_*1g*_ proper *R*$\bar{3}$*c* mode. But it is better to describe it as precession rotations than pure rotations^9^. This peak is well reproduced from our calculated Phonon Density of States (PDOS) (Fig. 5), obtained by Lattice Dynamic Calculation (LDC) using the shell model (UNISOFT code^32^, with the same settings as of previous study^9^). The PDOS for LMO has been studied experimentally by inelastic neutron scattering^33^ where many overlaps with the PDOS given in Fig. 5 can be recognized. It appears that the peak at 256–260 cm^−1^ contributes to the main PDOS activities in 60, 80 and 100 μC irradiated samples but reduces to sidebands in 20, 40 μC irradiated and un-doped samples. As discussed previously, the decrease of irradiation dose reduces microstrains and internal stress, which is equivalent to applying larger external pressure. This increase of external pressure at lower doses agrees well with the previous observations^34^ when this band diminishes at external pressures above 5.6 GPa. For our case of 40 μC, when this peak disappears, an internal pressure derived from Fig. 2(c) is 3.4 GPa. Taking into account that 7.8 GPa is a corresponding internal pressure for 100 μC sample, the 4.4 GPa difference between the two cases is what we need to apply as an external pressure on 100 μC sample to induce the disappearance of 256–260 cm^−1^ peak. It is commonly understood that, the increase of external pressure forces higher symmetry and reduces polarisation^4^. This is why the intensity of 256–260 cm^−1^ peak is small and diminishes at increasing external pressure (as *x* is sliding towards 0.50, Fig. 3(a,c)). This behaviour of a Raman mode is frequently referred to as belonging to rhombohedral distortion related soft modes^9,35^. The same point of view may be useful in explaining the intriguing activities found in Ca-doped LMO^36^. In this study, the two peaks at 235 and 435 cm^−1^ are expressing the blue shifts as temperature decreases. Because a thermal volume compression ΔV/V ≈ 0.45% is expected for 200 K decrease of temperature, a corresponding external pressure ≈ 1.0 GPa is deduced from Fig. 2(c). So, the blue shifts in Ca-doped LMO^36^ may be explained in term of increasing oscillator strengths due to stress.

It follows from Fig. 5 that the peaks at 486–489 cm^−1^ also express the similarity to the ones at 256–260 cm^−1^. Their positions vary only a little and do not seem to depend on irradiation dose, while their activities dominate in 60, 80 and 100 μC samples but decrease for 20, 40 μC and the un-doped ones. The same activities are also exhibited by the peaks at 638–644 cm^−1^, but with clear blue shifts on increasing dose. Recall that the similar peaks were identified as *E*_*g*_ pure oxygen bending vibrations (510 cm^−1^)^9^ and *E*_g_ out-of-phase stretching oxygen vibration (640 cm^−1^)^9^. Since the intensities of these peaks are strong, they are often observed in a number of cases (Table 2). They may also disappear under certain circumstances, *i.e*. below Curie temperature *T*_*c*_^10^, above Jahn-Teller transition temperature *T*_JT_ (~710 K)^11^, under magnetic field^37^, or in strongly doped cases^17,38^. Both 486–489 and 638–644 cm^−1^ bands couple with Jahn-Teller distortion of O_6_ cages^13^ but the assigned oxygen vibrations are still in question^8,39^. It is commonly agreed that both are one phonon modes, attributed to elementary excitation caused by orbital ordering (*alias* orbitons), or orbitons coupled with phonons^16,21,40^. The blue shifts of both modes are reported for *R*$\bar{3}$*c* LMO thin films^20,41^. The shifts seen in our samples for 638–644 cm^−1^ peaks can be regarded as the blue shifts at the volume expansions. Intuitively, the increase of external pressure increases Mn-O force constant *κ*, and proper Mn-O vibration frequency ν ($\nu =\sqrt{\kappa /\mu }/2\pi$, *μ* is a reduced mass). This consequently leads to blue shifts at volume compression, not at expansion as in our cases. Indeed, the pressure dependent blue shifts are often reported^34^. They occur even in cases where external pressure is not explicitly present, as for the oxygen excess compounds LaMnO_3+δ_ (Fig. 1 therein)^21^: the systematic blue shifts from 605 to 615 cm^−1^ are observed when δ increases from 0.0 to 0.07. This is because in the isothermal regime the pressure must increase when the volume compresses according to increasing δ. So far, the red shifts observed in our cases do not fall into this scheme. Naturally, it leads to the non-uniform impacts of microstrains, and argues for the fact that the microstrains may locally impose both volume expansion and compression. We can expect that the two effects are canceling each other macroscopically but at the microscopic level, where Raman is active, a small imbalance in favor of compression may induce blue shifts. So we can observe the blue shifts even in overall volume expansions. The data from our analysis confirm this scenario: the TCH strains *ε*_*θ*_ are twice as large as the lattice strains *ε*_*a*_, so the local compression of volume may be twice larger than the global average volume expansion.

As also depicted in Fig. 5, the peaks observed in a range 544–563 cm^−1^ (that split from a broad feature around 500 cm^−1^) behave nearly in the same manner as the ones at 638–644 cm^−1^. These peaks were not usually resoluble in the previous studies^9,10,19,20^ but the similar features are seen in the lower *Pnma* space group, such as in La_1−x_Ca_x_MnO_3-δ_ (555 cm^−1^, *B*_3g_(2) out-of-phase bending^17^), CaMnO_3_ (564 cm^−1^, *B*_3g_(2) symmetric breathing^42^, La_0.7_Sr_0.3_MnO_3_ (562 cm^−1^, *A*_*g*_ in-phase stretching^43^). The absence of these peaks in higher symmetry (or at high pressure^34^) directly links them to stress: the disappearance at lower doses is caused by the decrease of polarisation as symmetry increases. So, we suggest that the significant blue shifts of 544–563 cm^−1^ bands at increasing irradiation doses may also be explained by the non-uniform impacts of microstrains. A result of linear fitting yields ν(cm^−1^)  = 569.2−2.413 × *P* (with *P* is pressure in GPa). A slope of this equation agrees quite well with the ones retrieved from the data previously published^34^ for the same peaks. Since these peaks are clearly resoluble only when dislocations caused by irradiation increases, they should be assigned to a forbidden mode which is related to anti-symmetric vibrations of O_6_ cages.

Another scattering feature directly related to stress is a small peak within 662–667 cm^−1^ that is observed only in a few cases (Table 2). This peak corresponds to minor activity in PDOS of all irradiated samples but is a main activity in the un-doped one (Fig. 5). A similar feature has been reported previously for LMO under external pressure: above 3.0 GPa^34^ (or 7.0 GPa^4^) there appears a new band at around 680 cm^−1^ whose intensity grows with increasing pressure. This behaviour corresponds very well to the disappearance of a shoulder peak at around 665 cm^−1^ in our spectra, this peak cannot be seen in the spectra of 60, 80, and 100 μC as-prepared samples (front sides) (Fig. 4(d)) but is presented quite clear in the un-doped sample, 40 μC as-prepared and some sintered samples (front sides) and most back sides of as-prepared samples. As 3.0 GPa is equal to a difference between the derived pressures for 100 and 60 μC samples (Fig. 2(c)), the samples occur at lower doses (therefore at higher external pressures than 3.0 GPa) are approaching the occurrence of this new resonance band. According to the previous studies^4,34^ this peak is caused by the co-existence of undistorted octahedra (with *x* = 0.5) that are formed at high pressure. The occurrence of undistorted octahedra is necessarily associated with local changes of symmetry of connecting oxygen sites between two neighbouring MnO_6_ octahedra. This situation is similar to that of YMnO_3_ in *P6*_3_*cm* space group, where two apical oxygen atoms possess different site symmetry than that of the rest, so their displacements along axis *z* were assigned to a most pronounced resonance band observed at 681 cm^−1^ in YMnO_3_^44^. For LMO, a peak close to our 662–667 cm^−1^ has been reported^13^ at 655 cm^−1^ when the excitation wavelengths were shorter than 351 nm (energy greater than 3.53 eV) and was interpreted as a parent one phonon mode of a two phonon seen at 1300 cm^−1^. It couples strongly with a charge-transfer gap at 4.4 eV and is assigned to a vibrational oxygen breathing mode^13^. A peak at around 670 cm^−1^, whose intensity depends strongly on laser focus region, is also reported for rhombohedral La deficit structure La_0.91−δ_Mn_1-δ_O_3_^10^. Interestingly, this peak can also be seen in the orthorhombic La deficit La_1−δ_Mn_1−δ_O_3_ compounds. So its presence does not seem to depend on space group symmetry but rather relates to local deformations associated with one of oxygen sites and couples with uni-axial motions of this site along one octahedral axis (*e.g*. a rhombohedral axis [101]_*r*_). Therefore, the symmetric oxygen breathing mode appears as a reasonable assignment for the 662–667 cm^−1^ shoulders. The symmetry also explains why they become stronger when symmetry increases in high pressure.

Lastly, we discuss the two weak features observed in most of our samples, to which the previous studies seem not to pay enough attentions: the clear peaks at 320–328 cm^−1^ and the minor shoulders at 407–419 cm^−1^ (missing for the back sides). For comparison, we remind that, peaks in the same frequency range (320–328 cm^−1^) are reported for *Pnma* LMO (identified as *B*_*3g*_ in-phase *z* rotation mode)^8^, *R*$\bar{3}$*c* LMO (*E*_*g*_ pure Mn vibration mode)^9,18,45^, and for both orthorhombic and rhombohedral La deficit LMO^10^. The peaks at lower frequencies (but close to 320 cm^−1^) are seen in some doped cases, *e.g*. in the Na-doped LMO^46^. On the other hand, a peak treated as rotational mode^13^ is presented at 448 cm^−1^. A peak assigned to an *E*_*g*_ mode also appear for Na-doped LMO^46^ at 436.6 cm^−1^, for Sr, Zn-doped LMO^35^ at 427 cm^−1^. For both bulk and thin films LMO^45^ the similar peaks can be observed at around 425 cm^−1^. The La deficit LMOs represent the cases where peaks can be found at ~420 cm^−1^ in both orthorhombic and rhombohedral samples^10^. Since both features in our cases, 320–328 and 417–421 cm^−1^, show no clear dependence on irradiation, it will be naturally to suppose that these peaks are not related to rhombohedral distortions of O_6_ cages but to other kinds of displacements of atoms. Indeed, the results from our simulation show that there is a uni-axial translation-like movement of fixed O_6_ cages. There are two main directions, one is along a line bisecting two neighbouring O…O atoms (310–330 cm^−1^) and another is along one Mn-O bond (400–425 cm^−1^). A circular polarisation of La atoms may contribute to both these resonances too. Such kind of modes, of course should not be changed among the compounds, and indeed they are observed in different samples and symmetries.

## Methods

### Preparation of bulk materials

The LMO bulk samples were prepared by using a sol-gel citrate method with nitrate salts of lanthanum and manganese (0.5 M La(NO_3_)_3_ and 0.5 M Mn(NO_3_)_2_ solutions) as initial precursors. The stirred mixtures of equal amounts from each of these solutions were heated at 70 °C, and in continuous stirring the citric acid (1 M solution) was slowly added to maintain the pH between 3 and 5, until the white gels appeared. After drying, the gels were pre-sintered at 500 °C for 4 hours, and the obtained dark powder was ground in open air, and pressed into the disks of diameter 10 and thickness 1 mm under a pressure of 4500 kg/cm^2^. The final product was sintered at 1200 °C for 40 hours in Ar atmosphere, resulting in the un-doped bulk LMO samples.

### Irradiation by heavy-ions

The irradiated samples were obtained by subjecting the raw LMO disks to the LaO^−^ ion beam, produced from a Cesium Enhanced Negative Ion Sputter Source (SNICS II) and accelerated to desired energy by an electrostatic accelerator (Pelletron 5SDH-2). The ion beam energy was adjusted to allow a penetration depth of around 100 μm into the bulk targets. The total charges the beam imposed on the samples are 0, 20, 40, 60, 80, and 100 μC, which imply the real bulk concentrations of less than 0.05%. While the real distribution of ions in the whole sample is unknown and probably differs from case to case, we can safely consider at the first estimate that the distribution is Gaussian upon the implantation depth. This implies for our case of the thick discs that at the thin surface layers (where techniques like X-ray and Raman are active) the concentration is constant and the distribution is homogeneous in average. Recall that the accelerator in use is a Van de Graaff type accelerator, where the energy *E* (in MeV) of accelerated ions can be estimated empirically from the terminal voltage *V*_π_ (in MV) and charge *q* of ions as *E* = V_π_(*q* + 1). Therefore, the LaO^−^ ions at maximum 1.7 MV will attain maximum output energy of 3.4 MeV, which practically allows them to penetrate through thick layers of bulk materials. Thus, this type of accelerator is suitable for doping the bulk samples, unlike the early types which are equipped with low energy beams and can be used only for doping surfaces or thin films^47^.

### Characterization measurements

The X-ray diffractograms were obtained on Bruker D5005 diffractometer with CuK_α_ radiation (λ = 1.54056 Å), for 2*θ* from 20 to 80° at a step width of 0.03°. The Raman scattering measurements were performed at room temperature in backscattering geometry by using a He–Ne excitation laser of wavelength 632.8 nm. For each sample the X-ray and Raman scattering spectra were recorded for the front sides, which face towards the ion beam, and the back sides, which are attached to the substrate. Furthermore, to examine the structural changes after implantation (also to rule out the possible laser annealing effect during Raman experiments) we annealed all samples again at 500 °C for 8 hours in Ar and recorded the X-Ray and Raman spectra correspondingly.

### DFT modeling

The calculation was processed with Local Density Approximation (LDA) of the following settings (CASTEP code^48^): spin-polarized configuration, energy cut-off 380 eV, *k*-point set 3 × 3 × 2 (Monkhorst-Pack grid), convergence 5.0 × 10^−7^ eV/atom, ultra-soft potential, plane wave basis with LDA + U correction (U = 6.5 eV) for Mn *d-*electrons. Only diagonal elements of stress tensor are selected and non-zero. The starting model structure is considered as stress free. The symmetry is *R*$\bar{3}$ with antiferromagnetic spin configuration.

## Data Availability

X-ray, Raman and DFT data are available upon request. Correspondences should be addressed to HNN.
